# Atrial Fibrillation in Transthyretin Amyloid Cardiomyopathy: A Marker of Disease Severity but Not an Independent Predictor of Mortality

**DOI:** 10.7759/cureus.101917

**Published:** 2026-01-20

**Authors:** Carlos M Penate, Amalia Peix, Aylen Perez, Kenia Padron, Andrew S Dzebu, Carlos Fonseca, Jesus Rojas-Velazquez, Roxana Pazmino, Fernando Barba, Orlando Henriquez Italin

**Affiliations:** 1 Cardiology, Instituto Nacional de Enfermedades Respiratorias, Mexico City, MEX; 2 Nuclear Cardiology, Instituto de Cardiologia y Cirugia Cardiovascular, Havana, CUB; 3 Echocardiography, Instituto de Cardiologia y Cirugia Cardiovascular, Havana, CUB; 4 Cardiothoracic Centre, Ho Teaching Hospital, Ho, GHA; 5 Arrhythmia, Instituto de Cardiologia y Cirugia Cardiovascular, Havana, CUB; 6 Critical Care, Instituto de Cardiologia y Cirugia Cardiovascular, Havana, CUB; 7 Cardiology, Instituto de Cardiologia y Cirugia Cardiovascular, Havana, CUB; 8 Cardiology, Hermanos Ameijeiras Hospital, Havana, CUB

**Keywords:** amyloidosis, atrial fibrillation, cardiomyopathy, prognosis, transthyretin

## Abstract

Background and objective

Atrial fibrillation (AF) is the most common arrhythmia in patients with transthyretin amyloid cardiomyopathy (ATTR-CM), although its prognostic role remains uncertain. There is substantial evidence regarding mortality when AF coexists with heart failure (HF). However, this association is not well established in patients with ATTR-CM. This study aimed to assess whether AF was an independent predictor of all-cause mortality in ATTR-CM.

Methods

A total of 22 patients with confirmed ATTR-CM were followed (mean follow-up: 18.2 ± 6.8 months). The association between AF and all-cause mortality was evaluated using Cox proportional hazards modeling.

Results

The cohort had a mean age of 72 years (AF: 75.0 ± 5.5 years vs. no AF: 69.0 ± 8.7 years), and 81.8% of patients were men. Nine (41%) patients died during follow-up; among these, six (67%) had AF. AF was present in 11 (50%) of patients. AF was associated with more advanced disease, including a higher proportion of ATTR stage III (45.5% vs. 18.2%; p = 0.14) and New York Heart Association (NYHA) class III-IV symptoms (81.8% vs. 54.5%; p = 0.13). AF was not associated with all-cause mortality in the unadjusted analysis (hazard ratio (HR) 2.12; 95% confidence interval (CI): 0.53-8.52) or after adjustment for age, ATTR stage III vs. I-II, and left ventricular global longitudinal strain (LVGLS) > −15% (HR: 0.90; 95% CI: 0.12-6.98). In contrast, ATTR stage III vs. I-II remained an independent predictor of mortality (HR: 7.05; 95% CI: 1.55-31.98).

Conclusions

Patients with ATTR-CM carry a high mortality burden. AF is a common rhythm disorder in this population and appears to represent a more advanced disease phenotype rather than an independent predictor of all-cause mortality. These findings suggest that AF may function as a marker of disease progression and a valuable element for clinical stratification.

## Introduction

Cardiac amyloidosis (CA) is an infiltrative cardiomyopathy caused by the accumulation of misfolded proteins forming amyloid fibrils in the myocardial extracellular space, leading to restrictive physiology, heart failure (HF), and cardiac arrhythmias [1]. Atrial fibrillation (AF) is the most common rhythm disorder among patients with transthyretin amyloid cardiomyopathy (ATTR-CM), with a prevalence of greater than 50% [2]. In patients with ATTR-CM who have AF, amyloid infiltration of the atria results in impaired mechanical contraction, elevated filling pressures, a higher risk of thrombosis, and worsening HF [3-5]. The prognostic value of AF when it coexists with HF is well recognized [6]. However, its effect on mortality in ATTR-CM remains unclear. This gap in evidence underscores the need to better characterize the clinical importance of AF in patients with ATTR-CM.

## Materials and methods

Study design and population 

A prospective cohort study was conducted by following 22 patients with confirmed ATTR-CM (mean follow-up: 18.2 ± 6.8 months) at the Institute of Cardiology and Cardiovascular Surgery, Cuba, between 2021 and 2023. All eligible patients with complete follow-up information were included in the analysis. Race/ethnicity was obtained from the medical record at the index visit. Categories were recorded as White, Black, or Mixed, as documented in the medical record. Given the small cell counts, race/ethnicity was reported descriptively and not included in the adjusted models. Patients with a baseline estimated glomerular filtration rate (eGFR) below 30 ml/min/1.73 m², including those receiving chronic dialysis, were excluded a priori.

Diagnosis of ATTR-CM was established noninvasively in accordance with current European Society of Cardiology (ESC) criteria [1]. Noninvasive diagnosis required echocardiographic and/or cardiac magnetic resonance findings consistent with CA, together with grade 2 or 3 myocardial uptake on technetium-99m hydroxymethylene diphosphonate (99mTc-HMDP) scintigraphy with SPECT, and the absence of a monoclonal protein, confirmed by a serum free light-chain assay and serum and urine immunofixation electrophoresis. Patients were assigned to stages according to the ATTR staging system (stages I to III) described by Gillmore et al. [7]. This staging system categorizes patients into three stages: stage I, N-terminal pro-B-type natriuretic peptide (NT-proBNP) ≤ 3000 pg/mL and eGFR ≥ 45 mL/min/1.73 m²; stage II, patients who do not meet criteria for stage I or stage III; and stage III, NT-proBNP > 3000 pg/mL and eGFR < 45 mL/min/1.73 m².

The diagnosis of AF was based on a 12-lead ECG, Holter monitoring, or pacemaker interrogation performed at (or before) the index visit, or by established history or paroxysmal, persistent/long-standing persistent, or permanent AF, as per guideline criteria [8]. AF status was classified as baseline or historical at enrollment and treated as a time-fixed exposure for the primary analyses; patients without AF at baseline who developed AF during follow-up were not reclassified, given the absence of protocolized continuous rhythm monitoring. Accordingly, our analysis evaluated prevalent AF at baseline rather than incident AF, and AF incidence during follow-up was not systematically estimated.

Echocardiographic measurements were obtained according to established standard recommendations. In patients with AF, Doppler and strain parameters were averaged over multiple cardiac cycles, typically three to five beats. Left ventricular global longitudinal strain (LVGLS) analysis was performed using a Philips EPIQ 7 ultrasound system with EPIQ 7.1.1 software and a 2.5-MHz phased array probe. LVGLS had a prespecified cutoff of −15%, with LVGLS > −15% indicating more advanced myocardial dysfunction, based on prior ATTR-CM literature and clinical interpretation. Rhythm at the time of image acquisition (sinus rhythm or AF) was recorded and reported across groups. All-cause mortality was defined as death from any cause.

The association between AF and all-cause mortality was evaluated using Cox proportional hazards analysis; for multivariate analysis, covariates were selected a priori based on clinical relevance and parsimony criteria appropriate for the limited number of events. Age, ATTR stage III vs. I-II, and LVGLS > -15% were included due to their well-established prognostic value in ATTR-CM and their role as markers of disease severity. These variables were intentionally selected to reduce overfitting and to prevent collinearity with other echocardiographic or clinical measures of hemodynamic impairment.

Eligibility criteria

Inclusion Criteria

The inclusion criteria were as follows: 1. Patients aged ≥18 years with a confirmed diagnosis of ATTR-CM. 2. Patients who provided written informed consent to participate in the study.

Exclusion Criteria

The exclusion criteria were as follows: 1. Presence of severe comorbid conditions limiting life expectancy to less than one year 2. Inability to complete the required diagnostic evaluations or follow-up. 3. Advanced chronic kidney disease, defined as an eGFR < 30 mL/min/1.73m^2^. 4. Pregnancy or breastfeeding. 5. Diagnosis of light-chain (AL) amyloidosis or refusal to participate in the study.

The study population consisted of all patients diagnosed with ATTR-CM at the institution during the predefined recruitment period who had clinical follow-up at the hospital. Given the low prevalence of ATTR-CM, no formal sample size calculation was performed, as all eligible patients within the study population were included in the analysis (n = 22). 

Ethical consideration

The Institutional Ethics Committee of the Institute of Cardiology and Cardiovascular Surgery, Havana, approved the study protocol (ICCCV-CEI-01/2021), and written informed consent was obtained from all participants.

Statistical analysis

Statistical analyses were performed using IBM SPSS Statistics 27.0 (IBM Corp., Armonk, NY) and Stata/MP 14.1 (StataCorp, College Station, TX). Qualitative variables were expressed as absolute and relative frequencies. Quantitative variables were expressed as mean ± standard deviation (SD) or median with interquartile range (IQR), according to distribution assessed by the Shapiro-Wilk test. Comparisons between groups were performed using the chi-square or Fisher’s exact test for qualitative variables and Student’s t-test or Mann-Whitney U test for continuous variables, as appropriate. To assess the association between predictor variables and the occurrence of the outcome of interest, a Cox proportional hazards regression model was used. In univariate analysis, covariates included recognized predictors of mortality in patients with ATTR-CM reported in the medical literature, as well as variables that showed statistically significant associations with mortality in the exploratory analysis.

Given the limited number of events (nine deaths), the multivariate analysis was prespecified to include a small set of clinically relevant covariates to reduce overfitting: age, ATTR stage III vs. I-II, and LVGLS > -15%. Because eGFR is a component of the Gillmore ATTR staging system, it was not included in the multivariable analysis to avoid collinearity/overadjustment. Given the limited event count, we prioritized parsimony. Associations derived from both modeling strategies were expressed as hazard ratios (HR), with their corresponding 95% confidence intervals (CI). Survival curves were estimated using the Kaplan-Meier method for the evaluated mortality predictors. The proportional hazards assumption was assessed using Schoenfeld residuals; no evidence of violation was observed (global test p = 0.55). Missing data were handled using complete-case analysis; no imputation was performed. Because analyses were exploratory and hypothesis-generating, no formal adjustment for multiple comparisons was applied, and p-values should be interpreted descriptively. A two-sided p-value < 0.05 was considered statistically significant.

## Results

The baseline clinical and demographic characteristics of the study cohort are presented in Table 1. Of the 22 patients with ATTR-CM, nine (41%) died during the follow-up, and six (67%) of these nine patients had AF. AF was present in 11 (50%) patients in the overall cohort. Regarding the AF subtypes, two (19%) patients had paroxysmal AF, three (27%) had long-standing persistent AF, one (9%) had subclinical AF, and five (45%) had permanent AF.

**Table 1:** Baseline clinical and demographic characteristics in patients with or without AF Continuous variables were compared using Student’s t-test or Mann-Whitney U test (z statistic), and categorical variables using Fisher’s exact test AF: atrial fibrillation; SD: standard deviation; HTN: hypertension; T2DM: type 2 diabetes mellitus; CKD: chronic kidney disease; AV block: atrioventricular block; NYHA: New York Heart Association; NT-proBNP: N-terminal pro-B-type natriuretic peptide; eGFR: estimated glomerular filtration rate; ATTR: transthyretin

Variable		With AF (n = 11)	Without AF (n = 11)	Test statistic	P-value
Age, year, mean ± SD		75.0 ± 5.5	69.0 ± 8.7	t = - 1.66	0.11
Sex, n (%)	Male	9 (81.8)	9 (81.8)	Fisher: -	1.00
	Female	2 (18.2)	2 (18.2)		
Race, n (%)	White	4 (36.4)	5 (45.5)	Fisher: -	0.66
	Black	4 (36.4)	2 (18.2)		
	Mixed	3 (27.2)	4 (36.3)		
Symptoms, n (%)	Dyspnea	5 (45.5)	6 (54.5)	Fisher: -	0.56
	Fatigue	4 (36.4)	4 (36.4)		
	Syncope	1 (9.1)	0 (0)		
	Chest pain	1 (9.1)	1 (9.1)		
Comorbidities, n (%)	HTN	5 (45.5)	8 (72.7)	Fisher: -	0.19
	T2DM	2 (18.2)	3 (27.3)	Fisher: -	0.61
	CKD	7 (63.6)	4 (36.4)	Fisher: -	0.21
ECG, n (%)	Low voltage	7 (63.6)	4 (36.3)	Fisher: -	0.21
	AV blocK	4 (36.4)	2 (18.2)	Fisher: -	0.33
NYHA functional class, n (%)	I	2 (18.2)	1 (9.1)	Fisher: -	0.13
	II	0 (0.00)	4 (36.4)		
	III-IV	9 (81.8)	6 (54.5)		
Biomarkers	NT-proBNP, pg/mL, median (Q1-Q3)	4590 (2992-6178)	2605 (1306-3864)	z = - 2.07	0.04
	Troponin I, ng/mL, median (Q1-Q3)	201 (97-305)	163 (68-258)	z = - 0.56	0.55
	eGFR, ml/min/1.73m^2, ^mean ± SD	56 ± 16.8	66 ± 18.8	t = 1.37	0.18
ATTR stage, n (%)	Stage I	1 (9.1)	5 (45.5)	Fisher: -	0.14
	Stage II	5 (45.5)	4 (36.4)		
	Stage III	5 (45.5)	2 (18.2)		

Patients with AF were older (75.0 ± 5.5 vs. 69.0 ± 8.7 years; p = 0.11); sex distribution was similar between groups (82% male); and ethnicity distribution did not differ significantly (p = 0.66). Dyspnea was the most frequent symptom, with a similar distribution (45.4% vs. 54.5%). Arterial hypertension was the most common comorbidity (45.5% vs. 72.7%); the difference was not statistically significant. Regarding electrocardiographic findings, low voltage was more frequent among AF patients (63.6% vs. 36.3%; p = 0.21), and atrioventricular block was also more common (36.3% vs. 18.2%; p = 0.33), although neither difference reached statistical significance. Among biomarkers, elevated NT-proBNP concentrations were significant in the AF group (p = 0.04), while troponin I (p = 0.55) and eGFR (p = 0.18) did not reach statistical significance. A greater proportion of patients with AF presented with NYHA class III-IV symptoms (81.8% vs. 54.5%; p = 0.13). ATTR stage III was also more frequent in the AF group (45.5% vs. 18.2%; p = 0.14). Although neither reached statistical significance.

Echocardiographic parameters were similar between groups for left ventricular ejection fraction (LVEF), left ventricular end diastolic diameter (LVEDD), left ventricular mass (LVM), stroke volume (SV), grade of diastolic dysfunction, and left atrial volume index (LAVI) (all p ≥ 0.05). Furthermore, left atrial thrombus was detected exclusively in the AF group (18.2% vs. 0%; p = 0.07) (Table 2).

**Table 2:** Echocardiographic characteristics in patients with or without AF Continuous variables were compared using Student’s t-test or Mann-Whitney U test (z statistic), and categorical variables using Fisher’s exact test AF: atrial fibrillation; LVEF: left ventricular ejection fraction; SD: standard deviation; LVEDD: left ventricular end diastolic diameter; LVM: left ventricular mass; SV: stroke volume; LAVI: left atrial volume index; GLS: global longitudinal strain; LA thrombus: left atrial thrombus

Characteristics		With AF (n = 11)	Without AF (n = 11)	Test statistic	P-value
LVEF, %, mean ± SD		53.2 ± 13.3	52.0 ± 15.5	t = - 0.19	0.85
LVEDD, mm, median (Q1-Q3)		45.7 (39.3-52.0)	42.1 (37.9-46.3)	z = -1.22	0.22
LVM, g, mean ± SD		236.0 ± 85.3	197.8 ± 68.2	t = - 1.15	0.26
SV, mL, median (Q1-Q3)		66.8 (47.24-86.3)	74.0 (61.35-86.82)	z = - 0.69	0.49
LAVI, mL/m^2, ^mean ± SD		50.3 ± 15.8	43.0 ± 14.2	t = -1.07	0.29
LVGLS > -15%, n (%)		8 (75.0)	11 (100.0)	Fisher: -	0.05
LA thrombus, n (%)		2 (18.2)	0 (0)	Fisher: -	0.07
E/e', median (Q1-Q3)		19.9 (17.0-22.9)	16.9 (12.9-20.9)	z = - 1.32	0.19
Diastolic dysfunction, n (%)	Grade 1	0 (0)	4 (36.3)	Fisher: -	0.06
	Grade 2	5 (45.5)	2 (18.2)		
	Grade 3	6 (54.5)	5 (45.5)		

The AF subtypes were observed across all ATTR stages (Table 3). In stage 1, the only documented episode was paroxysmal AF. Stage 2 included paroxysmal, longstanding persistent, and permanent AF. In stage 3, permanent AF was the most frequent. Given the small sample size, formal comparisons were not performed. They were considered exploratory and not modeled. 

**Table 3:** Distribution of AF subtypes across ATTR-CM stages (n = 11 patients with AF) AF: atrial fibrillation;; ATTR-CM: transthyretin amyloid cardiomyopathy

ATTR stage	Stage I (n = 1), n (%)	Stage II (n = 5), n (%)	Stage III (n = 5), n (%)
Paroxysmal	1 (100)	1 (20.0)	0
Longstanding persistent	0	2 (40.0)	1 (20.0)
Subclinical	0	0	1 (20.0)
Permanent	0	2 (40.0)	3 (60.0)

Table 4 summarizes the results of the Cox proportional hazards analysis evaluating prognostic factors associated with mortality. In the unadjusted model, AF was not associated with an increased risk of all-cause mortality (HR: 2.12, 95% CI: 0.53-8.52; p = 0.262), and this finding remained unchanged after adjustment for age, ATTR stage III vs. I-II, and LVGLS > -15%, remaining nonsignificant (HR: 0.90; 95% CI: 0.12-6.98; p = 0.92). Other variables demonstrated statistically significant associations with mortality, including NT-proBNP (HR: 1.003, 95% CI: 1.001-1.007; p = 0.016), troponin I (HR: 1.006, 95% CI: 1.005-1.010; p = 0.010), eGFR (HR: 0.93, 95% CI: 0.89-0.98. p = 0.04), ATTR stage III vs. I-II (HR: 6.15, 95% CI: 1.52-24.94; p = 0.011), LVEF (HR: 0.93, 95% CI: 0.87-0.99; p = 0.016), and LA thrombus (HR: 9.62, 95% CI: 1.36-68.3; p = 0.024). ATTR stage remained an independent predictor of mortality in multivariate analysis (HR: 7.05, 95% CI: 1.55-31.98; p = 0.011).

**Table 4:** Univariate and multivariate Cox proportional hazards regression analysis for all-cause mortality Multivariable model adjusted for age, ATTR stage, and LVGLS > -15% HR: hazard ratio; CI: confidence interval; NT-proBNP: N-terminal pro-B-type natriuretic peptide; eGFR: estimated glomerular filtration rate; ATTR: transthyretin; NYHA: New York Heart Association; LVEF: left ventricular ejection fraction; LVM: left ventricular mass; LVEDD: left ventricular end diastolic diameter; LVGLS: left ventricular global longitudinal strain; LAVI: left atrial volume index; LA thrombus: left atrial thrombus

Variables	Univariate analysis			Multivariate analysis		
	HR	95% CI	P-value	HR	95% CI	P-value
Atrial fibrillation	2.12	0.53-8.52	0.262	0.90	0.12-6.98	0.92
Age	1.01	0.93-1.09	0.85	0.97	0.85-1.10	0.61
NT-proBNP	1.003	1.001-1.007	0.016	-	-	-
Troponin I	1.006	1.005-1.010	0.010	-	-	-
eGFR	0.93	0.89-0.98	0.04	-	-	-
Low voltage	4.59	0.95-22.2	0.058	-	-	-
ATTR stage III vs. I-II	6.15	1.52-24.94	0.011	7.05	1.55-31.98	0.011
NYHA class	2.56	0.84-7.83	0.099	-	-	-
LVEF	0.93	0.87-0.99	0.016	-	-	-
LVM	1.06	0.99-1.07	0.122	-	-	-
LVEDD	1.01	0.93-1.10	0.772	-	-	-
LVGLS > -15%	1.34	0.17-10.73	0.78	0.28	0.02-4.00	0.35
LAVI (mL/m^2^)	1.03	0.96-1.05	0.87	-	-	-
LA thrombus	9.62	1.36-68.3	0.024	-	-	-
Grade of diastolic dysfunction	1.39	0.49-3.91	0.53	-	-	-

**Figure 1:**
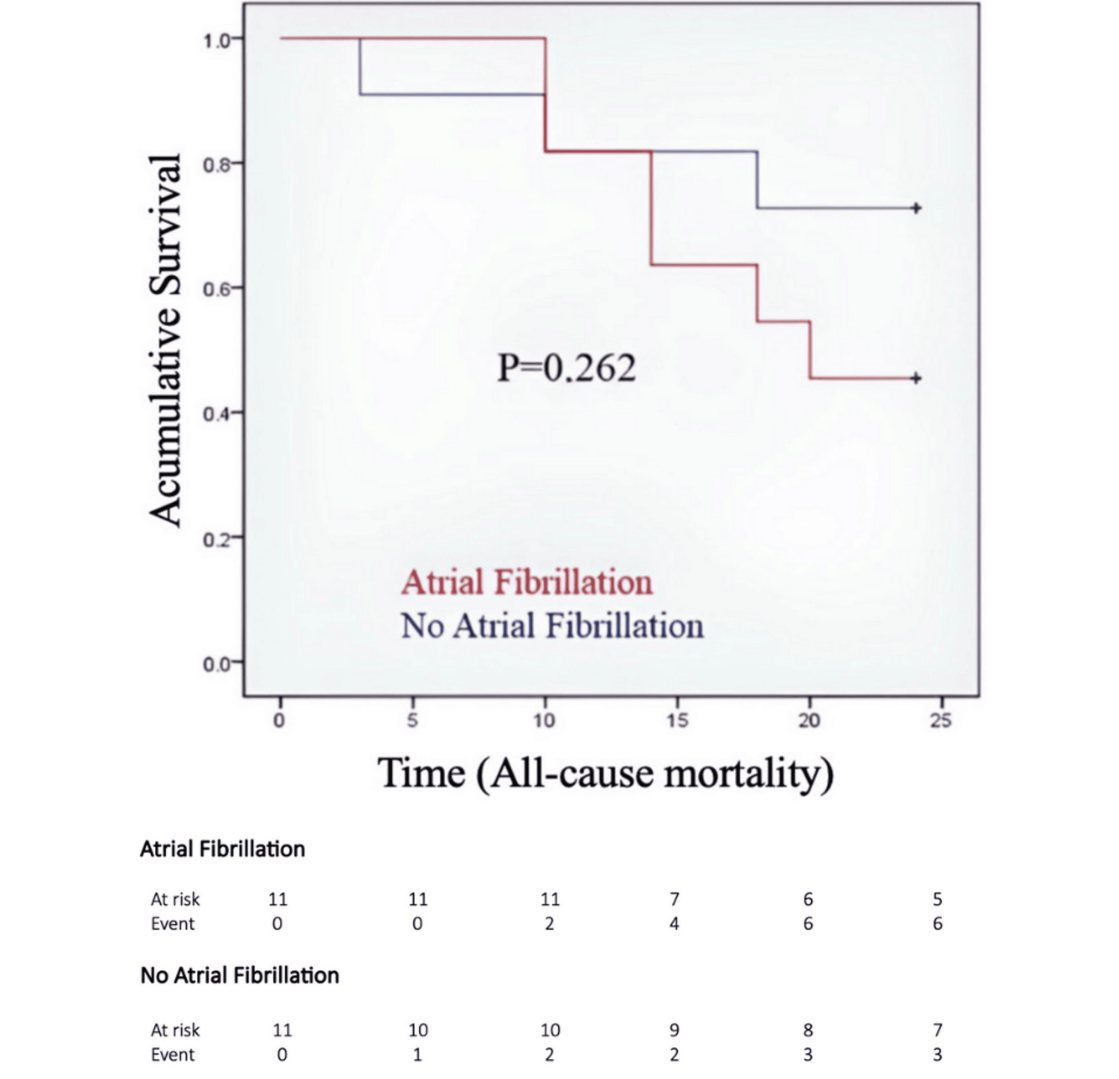
Kaplan-Meier survival curves according to atrial fibrillation status Survival curves were compared using the log-rank (Mantel-Cox) test

Figure 1 shows the Kaplan-Meier survival curves according to AF status. Patients with or without AF did not exhibit a statistically significant difference in overall survival (log rank p = 0.262). Median overall survival was not reached during follow-up. The Kaplan-Meier estimated survival at 25 months was 59.1% (95% CI: 36.1% - 76.2%). The restricted mean survival time (RMST) at 25 months was 17.6 months in patients without AF and 10.4 months in patients with AF (difference -7.2 months; AF minus no AF), reported descriptively given the small sample size.

## Discussion

In this cohort study, half of the patients with ATTR-CM had AF at enrollment, which is consistent with the high prevalence reported in contemporary series [2]. Our results suggest an apparent increase in mortality among patients with AF. However, this association was no longer statistically significant in the survival analysis using the Cox proportional hazards model (Figure 1). The overall mortality rate of ATTR-CM is markedly elevated, particularly among patients in stage III, which likely obscures any additional prognostic impact of AF [7,9]. These findings are consistent with previous studies. Witteles et al. [10] showed that AF was associated with mortality only in a minimally adjusted model that included age and sex. Similarly, Sanchis et al. [11] reported that AF lost its statistical significance after adjusting for age. Likewise, Longhi et al. [12] and Mints et al. [13] demonstrated that AF is more closely related to advanced disease than it is an independent predictor of mortality. CA is initially characterized by isolated diastolic dysfunction due to increased myocardial ventricular mass from amyloid infiltration, wall stiffness, and reduced SV [14].

Additionally, progressive infiltration of the atrial walls results in impaired mechanical atrial function, which, when combined with loss of effective atrial contraction inherent to AF, leads to markedly reduced atrial emptying and further elevation of filling pressure [15]. These alterations establish a substrate favorable for the development of HF [4,9]. Also, thrombus formation was seen even among patients receiving oral anticoagulation [5]. Furthermore, patients with AF exhibited higher proBNP levels; most were classified as ATTR stage III and presented advanced HF (NYHA III/IV). These findings indicated that AF represents a more advanced phenotype, with a markedly increased risk of HF decompensation. Therefore, AF should be regarded primarily as an indicator of disease progression, and its onset may necessitate reassessment of the clinical stage. 

Limitations of the study

This study has certain limitations. It involved a single-center cohort with a limited number of patients (n = 22) and a low number of outcome events, which contributed to the wide CIs observed in both analyses and the restricted number of covariates that could be included in the adjusted model. Although we adjusted for age, ATTR stage III vs. I-II, and LVGLS > -15%, residual confounding cannot be excluded, as additional prognostic markers could not be incorporated due to the limited number of events. AF subtype and duration may have been misclassified because ascertainment relied on routine clinical documentation, and the absence of protocolized continuous rhythm monitoring may have led to under-detection of subclinical or new-onset AF during follow-up.

Furthermore, patients did not have access to disease-modifying therapy, such as tafamidis. This lack of therapy was reflected by the high mortality observed, and limits comparisons with studies in which this medication was used. Therefore, we cannot exclude residual confounding due to untreated disease progression. Genetic testing was not systematically available for stratified analyses to distinguish hereditary from wild-type ATTR. Given the exploratory and hypothesis-generating nature of the analysis, no formal statistical adjustments for multiple comparisons were performed, and p-values should be interpreted as descriptive.

## Conclusions

Patients with ATTR-CM have a high mortality burden. AF is a common rhythm disorder in this population and seems to reflect a more advanced disease phenotype rather than serve as an independent predictor of all-cause mortality. These findings suggest that AF may act as a marker of disease progression and represent a valuable element for clinical stratification.
